# Transcranial direct current stimulation facilitates cognitive multi-task performance differentially depending on anode location and subtask

**DOI:** 10.3389/fnhum.2014.00665

**Published:** 2014-09-08

**Authors:** Melissa Scheldrup, Pamela M. Greenwood, Ryan McKendrick, Jon Strohl, Marom Bikson, Mahtab Alam, R. Andy McKinley, Raja Parasuraman

**Affiliations:** ^1^Arch Lab, Department of Psychology, George Mason UniversityFairfax, VA, USA; ^2^Neural Engineering Group, Department of Biomedical Engineering, The City College of New York of CUNYNew York, NY, USA; ^3^711th HPW, Warfighter Interfaces Division, Applied Neuroscience Branch, WPAFBWright-Patterson AFB, OH, USA

**Keywords:** tDCS, cognitive training, attention networks, multi-task

## Abstract

There is a need to facilitate acquisition of real world cognitive multi-tasks that require long periods of training (e.g., air traffic control, intelligence analysis, medicine). Non-invasive brain stimulation—specifically transcranial Direct Current Stimulation (tDCS)—has promise as a method to speed multi-task training. We hypothesized that during acquisition of the complex multi-task Space Fortress, subtasks that require focused attention on ship control would benefit from tDCS aimed at the dorsal attention network while subtasks that require redirection of attention would benefit from tDCS aimed at the right hemisphere ventral attention network. We compared effects of 30 min prefrontal and parietal stimulation to right and left hemispheres on subtask performance during the first 45 min of training. The strongest effects both overall and for ship flying (control and velocity subtasks) were seen with a right parietal (C4, reference to left shoulder) montage, shown by modeling to induce an electric field that includes nodes in both dorsal and ventral attention networks. This is consistent with the re-orienting hypothesis that the ventral attention network is activated along with the dorsal attention network if a new, task-relevant event occurs while visuospatial attention is focused (Corbetta et al., 2008). No effects were seen with anodes over sites that stimulated only dorsal (C3) or only ventral (F10) attention networks. The speed subtask (update memory for symbols) benefited from an F9 anode over left prefrontal cortex. These results argue for development of tDCS as a training aid in real world settings where multi-tasking is critical.

## Introduction

Many occupations of high importance to the nation (e.g., air traffic control, intelligence analysis, medicine) require long periods of training on complex multi-tasks. There is a clear need to facilitate such training. Early investigations into ways to shorten training times manipulated the relation between task load and cognitive load (Wickens et al., 2012). Recently, another potential method to speed task acquisition has been investigated: non-invasive brain stimulation (Clark and Parasuraman, 2014). One such brain stimulation technique is transcranial Direct Current Stimulation (tDCS) which applies a weak DC current to the scalp, thereby altering neuronal excitability in cortex (Bikson et al., 2009). Most previous research to date on the effects of tDCS on cognitive tasks has examined basic functions such as working memory and attention (reviewed in Jacobson et al., 2012; Coffman et al., 2014). Recently, interest has turned to use of tDCS to facilitate acquisition of cognitive tasks that are quite complex and capture aspects of work or everyday environments, viz., decision-making (Fecteau et al., 2007; Boggio et al., 2010), driving a complex route (Beeli et al., 2008), military threat detection (Clark et al., 2012; Falcone et al., 2012), and air traffic control (Nelson et al., 2014). Such findings raise the prospect that tDCS could facilitate multi-task training in real-world settings. However, there are several questions about use of tDCS with complex multi-tasks that need to be addressed.

First, it is important to determine the effect of tDCS in accelerating the *initial* phases of difficult multi-task cognitive training, before expertise is established. There are critical mechanisms of brain plasticity that are evoked early, and not late, in learning. In animals, expression of “immediate early genes” is the first genetic response to synaptic activity during learning and memory formation. One such gene, zif268 is important in (a) early but not later phases of learning (Maroteaux et al., 2014), and (b) the transition from short-term to long-term memory formation (Jones et al., 2001). Further, the role of attention appears to change over the course of training. Attention has an important role early in learning a task but that role is reduced as the task is mastered (Kelly and Garavan, 2005; Lewis et al., 2009; Strenziok et al., 2014). Considered together, this evidence argues for measuring effects of tDCS early in multi-task training while task-related memories are being formed.

Secondly, it is important to determine which brain regions to stimulate in order to facilitate multi-task training. Most of the previous tDCS literature has investigated effects of stimulating one brain region on one cognitive function. However, the weight of the evidence increasingly supports the view that cognition results from dynamic interactions between large-scale networks rather than from activity in isolated brain regions (Bressler and Menon, 2010). Further, there is evidence that tDCS increases functional connectivity in the dorsal attention network (Keeser et al., 2011)—a large-scale, resting state network specifically linked to cognitive training (e.g., Lewis et al., 2009; Strenziok et al., 2014). Resting state networks are defined by correlated spontaneous activity within spatially distinct cortical and subcortical regions in the absence of a task (Biswal et al., 1995; Greicius et al., 2009). This argues for considering known resting state networks in selecting tDCS montages (placement of anode and cathode) for tDCS to facilitate cognitive training.

In light of this, we sought to investigate effects of montages aimed at stimulating specific resting state networks on subtask performance in the initial learning of a multi-task typically mastered over weeks.

### tDCS alters neuronal activity

Following tDCS stimulation, polarization occurs both in the soma and synaptic terminals of pyramidal and non-pyramidal tract neurons (Stagg and Nitsche, 2011; Rahman et al., 2013), with cell compartment polarization being polarity specific with proximity to the anode or cathode. Studies examining effects of tDCS on motor cortex find that the cortical excitability is increased under the anode but decreased under the cathode (Nitsche and Paulus, 2000; Stagg et al., 2009). Studies examining effects of tDCS on cognitive tasks generally show heightened functionality with an anode over targeted cortex, with weaker or null effects when the cathode was over targeted cortex (Jacobson et al., 2012).

### tDCS influences complex task acquisition

To date, only a few studies have investigated effects of tDCS on real world-relevant cognitive multi-tasks—the focus of our research efforts. Decision-making was altered by bilateral stimulation of the dorsolateral PFC (dlPFC) (Fecteau et al., 2007). A “threat detection” task developed to train military personnel to detect concealed objects benefited from placing the anode over either F10 or P4 (contralateral shoulder cathode). Those locations were selected based on fMRI BOLD signals before and after training to an “expert” level of performance. Anodal tDCS to both sites improved threat detection performance compared to sham (Clark et al., 2012). A replication of this study by our group used signal detection theory to demonstrate that performance improvements on this task were attributable to increased perceptual sensitivity and not to changes in response bias. Performance improvements were retained for at least 24 h after stimulation (Falcone et al., 2012). This small literature, while suggesting that tDCS may be useful in training in real-world settings, has been limited by studies that: (a) used tasks that were mastered relatively quickly; (b) did not separately assess subtasks of multi-tasks; (c) did not compare a range of stimulation montages.

### Role of resting state networks in cognitive training

In concert with the growing recognition that cognition emerges from core large-scale brain networks (Bressler and Menon, 2010), recent evidence suggests that certain resting state networks have an important role in cognitive training. Corbetta et al. (2008) argued from a large body of neuroimaging work that the dorsal attention network plays a role in focusing visuospatial attention on relevant events while ignoring irrelevant events by suppressing the ventral network to prevent reorienting. Although this hypothesis was developed based on evidence from attention tasks, it has recently been found to be relevant to training effects. Nodes in the dorsal attention network [including intraparietal sulcus (IPS) and superior parietal cortex (SPC)] selectively undergo structural and functional changes following both motor and cognitive training (Draganski et al., 2004; Olesen et al., 2004; Dahlin et al., 2008; Lewis et al., 2009; Scholz et al., 2009; Takeuchi et al., 2010; Strenziok et al., 2014). This evidence suggests that focusing attention in the face of distraction is important in successful training, regardless of the specific training task. The right hemisphere ventral attention network [with nodes in right ventral frontal (VFC) and right temporo-parietal junction (TPJ)] is important for redirecting focused attention (Corbetta et al., 2008). The ventral attention network is claimed to be suppressed when attention is focused, but transiently activated along with the dorsal network when attention is redirected in response to a task-relevant event (Corbetta et al., 2008). Consistent with that hypothesis, changes in nodes of the ventral attention network have also been observed following training (Lee et al., 2012; Prakash et al., 2012). Relevant to the present study, tDCS selectively alters functional connectivity in resting state networks (Polanía et al., 2011), including the dorsal attention network (Keeser et al., 2011).

Considered together, there is growing evidence that both dorsal and ventral attention networks play an important role in cognitive training, suggesting that tDCS could be effective in facilitating training to the extent that it heightens activity in those networks.

### Rationale and hypotheses

To assess effects of tDCS on the initial phase of multi-task training, we tested hypotheses on the effect of stimulating specific resting state networks during the first 30 min of training on component subtasks of a multi-task typically learned over weeks. We selected the Space Fortress (SF) task (Mane and Donchin, 1989) for the following reasons: (a) SF is a complex task requiring integration of spatial, motor, and executive function demands, making it a good simulation of real-world multi-tasks; (b) SF is demanding and typically learned over a number of weeks, making it a good simulation of tasks that require considerable time to master; (c) SF subtasks provide separate measures of different task demands, allowing hypotheses to be tested about the relation between site of stimulation and performance on subtask measures during training; (d) we recently found that SF training altered functional connectivity between the dorsal attention network and temporal association cortex (Strenziok et al., 2014), consistent with other findings of altered connectivity involving the dorsal network following cognitive training (Lewis et al., 2009; Takeuchi et al., 2010).

We compared stimulation montages that were selected based on the neurocognitive hypothesis of attentional reorienting (Corbetta et al., 2008) and modeled to predict regional brain current flow (Datta et al., 2009). Overall, we hypothesized that tDCS would exert selective effects on subtasks of the multi-task very early in training. Specifically, we hypothesized that tDCS aimed at either right or left IPS in the dorsal attention network (C4, C3, respectively), associated with focusing visuospatial attention, would facilitate learning ship control and ship velocity subtasks that require attention to be focused on flying the ship. We also hypothesized that tDCS aimed at ventral prefrontal (F10) and TPJ (C4) in the right hemisphere ventral attention network, associated with redirecting attention, would facilitate learning of speed and points subtasks. Those two subtasks require redirection of attention from flying the ship toward symbols on the screen. As both dorsal and ventral networks are claimed to be active if a task-relevant event triggers reorientation while attention is focused (Corbetta et al., 2008), we predicted that the strongest benefits on redirecting attention from control and velocity to speed and points subtasks would be when both dorsal and ventral networks are simultaneously stimulated by right parietal tDCS (C4) inducing electrical fields over both IPS and TPJ. We tested our hypotheses by random assignment to sham or active right or left hemisphere ventral prefrontal (F9, F10) or parietal stimulation (C3, C4) during the first 30 min of a 45 min training session on the SF task. We acknowledge that uncertainties exist in the relation between the tDCS-induced electrical field and the brain regions and networks activated as a consequence and our approach was based on above-reviewed evidence of the role of the dorsal and ventral attention networks in training effects.

## Methods

### Participants

A total of 100 undergraduate students were recruited to participate in this study. Participants were recruited through George Mason University's participant recruitment system and students received course credit for their participation. All participants were right-handed, based on self-report and on experimenter observation of the hand used to fill out the questionnaires. Participants had normal or corrected to normal vision (20/30) based on the Rosenbaum pocket screener, no history of psychiatric or mental illness based on a questionnaire, and were not taking psychoactive medication. All participants provided written consent with procedures approved by the IRB of George Mason University. Participants completed a questionnaire to report the average number of hours spent per week playing different types of video games, including first-person shooter-type games. This is important in light of evidence that people who frequently play first-person shooter-type games have enhanced attentional abilities (Green and Bavelier, 2012).

### Apparatus

The laboratory testing room was equipped with a computer with a Windows operating system and a 17 inch monitor. A joystick was operated by the right hand and a three-button mouse was operated by the left hand. Participants were seated at an average distance of 24 inches from the monitor but were allowed to adjust the distance and chair position to their comfort.

### The space fortress task

**Figure 1:**
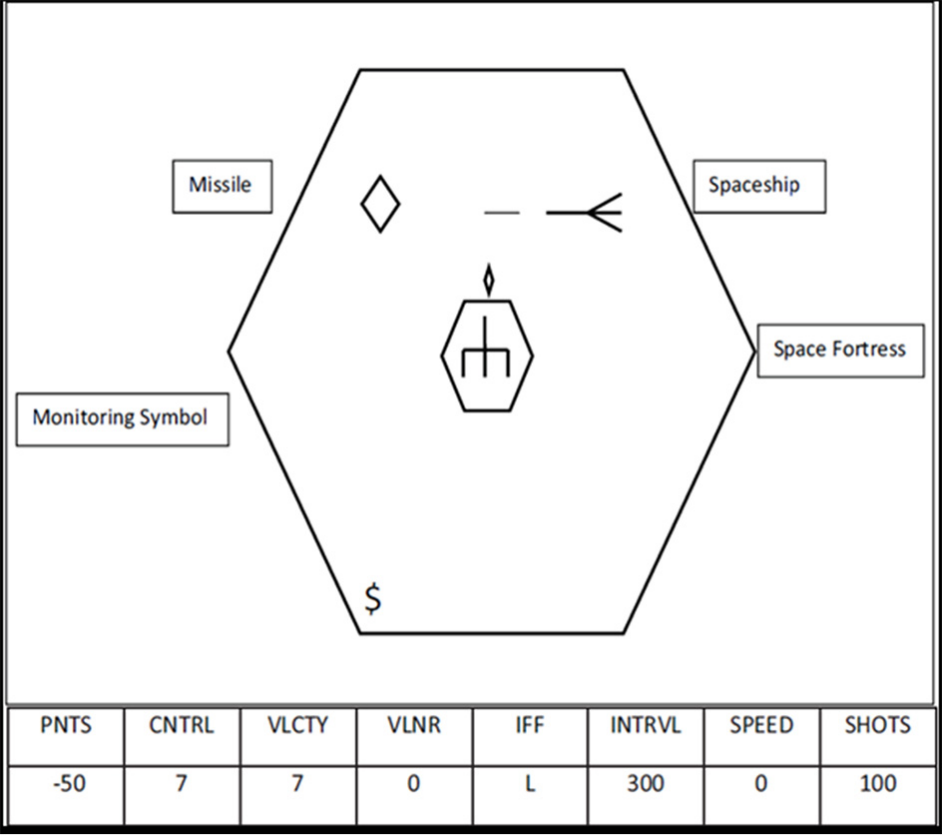
**Diagram of the Space Fortress game, figure originally published in Blumen et al. (2010)**.

The Space Fortress task was developed by cognitive psychologists to study learning of complex and important real-world tasks. We briefly describe this complex task here, but details of design and scoring can also be found in Gopher et al. (1989, 1994). The goal of this game is to destroy the Space Fortress in the center of the screen (Figure 1) while dealing with “friend” and “foe” mines and monitoring resources (i.e., number of missiles). The player uses a joystick in the right hand to navigate their ship while firing missiles at the Space Fortress, which becomes vulnerable to destruction after being hit by missiles 10 times with at least 250 ms between any two hits. After the Space Fortress becomes vulnerable, the player must destroy the Fortress by firing two missiles in quick succession (less than 250 ms between shots). The vulnerability of the Fortress is reset to 0 if any two shots are made within 250 ms of each other prior to becoming vulnerable or if the player's space ship is destroyed. Each trial was one game lasting 3 min. Performance measurements include total score, which is the sum of 4 subscores, each assessing a different component of the game; control subscore, velocity subscore, speed subscore, and points subscore.

#### Control subscore

To effectively destroy the Space Fortress, players must maintain control of the ship in the frictionless environment of space, avoiding large joystick movements which will cause the ship to accelerate and fly uncontrollably. There is no braking mechanism for slowing forward momentum. In order to maintain or regain control of the ship, players must use small joystick movements to aim the ship in the direction of flight and apply minimal thrust. To slow, stop, or reverse course, players must rotate the ship in the opposite direction and apply very light thrust. To achieve a high control subscore, players are instructed to fly their ship in a clockwise pattern around the Space Fortress while staying between two concentric hexagons visible around the Space Fortress. The control subscore increases continuously when the ship is within the two hexagons (full points) or on the screen outside the hexagons (half points), and decreases when the ship flies off the screen (ship wraps) or hits the inner hexagon surrounding the Space Fortress.

#### Velocity subscore

Velocity score is continuously increased while the ship is being flown at a low velocity but subtracted when the ship accelerates. Because of the frictionless environment and lack of a braking mechanism, once the ship accelerates the player must perform the same ship rotation maneuver described above. The correct amount of applied thrust will slow and/or stop the ship from moving, but too much thrust will send the ship flying at a high velocity in the opposite direction.

#### Speed subscore

Throughout each game, mines appear on the screen at irregular intervals. Prior to the start of each game, three letters are displayed indicating mines that are considered “foe” mines for that game. During the game, mines appear on the screen and a letter is displayed along the bottom of the screen (under the heading IFF—identify friend or foe). If the displayed letter was included in the 3 letters previously displayed, then the current mine is a “foe” mine, all other mines are “friend” mines. Friend mines can be shot and the damage is transferred to the Space Fortress. Foe mines need to first be identified by double clicking the mouse in the player's left hand before it can be destroyed. If foe mines are not destroyed, or friend mines are misidentified as a foe, both mines seek out the player's ship and damage it. The speed subscore is increased by quickly and correctly identifying and destroying the mines, but is reduced for misidentification or for a long reaction time before identification.

#### Points subscore

The points subscore is increased every time the player destroys a mine, damages or destroys the Space Fortress, or selects bonus points. The points subscore is decreased when the player fires a missile while they have none left, when their ship is hit, or when their ship is destroyed by either the fortress or a mine. In addition to trying to destroy the Space Fortress and the mines, another monitoring task affects points—resource bonuses. Periodically, symbols appear on the screen. After the second “$” appears, the player can indicate with the mouse if they would rather add 50 more missiles to their stock or have their points subscore increased by 100.

#### Training schedule

The training schedule was designed to promote task acquisition in one session. Previous work showed that SF learning benefits from “emphasis change” instructions—to play the whole game but periodically change emphasis to focus on different subcomponents of the game (Gopher et al., 1989). Before the application of tDCS electrodes, participants were given a 20 min Powerpoint presentation describing the basic rules and strategies of this complex game. After the presentation, participants took a 20 question quiz to evaluate their understanding of the rules. Participants were allowed to return to the presentation after an incorrect response and correct their mistake until they provided the correct answer, after which they were allowed to move on to the next question. In order to maintain a level of motivation, participants were informed that throughout the training there would be three performance assessments and that for each assessment, the participant with the highest total score would win $30, giving a total possible $90 motivational incentive.

***Aiming task***. After demonstrating an understanding of the rules, participants completed an “aiming task” game to assess their basic aiming and joystick control. The aiming task (Gopher et al., 1989) is a 3 min game with a stationary space ship that the player must rotate to aim and shoot missiles with the goal of destroying a mine. Mines appear on the screen in a random location order and remain for 10 s or until they are hit by a missile. The total score for this task was derived from adding the points and speed subscores. Because the spaceship is stationary, ship control and velocity subtasks are not required. After the aiming task, a feedback screen displayed total score, points subscore, and speed subscore. The total score was read to the participant by the experimenter. Previous studies (Gopher et al., 1989; Mane et al., 1989) have used scores from a 3-game aiming task as exclusion criteria, assuming that a certain baseline level of aiming and joystick control is necessary for training to be effective. As our design had a condensed training schedule with a 1-game aiming task, we did not exclude participants based on their aiming task score. However, a comparison of the stimulation montage groups on this task showed no group differences (see Results).

***Pre-stimulation baseline game***. After the aiming task, participants performed a pre-stimulation game (Game 1). During this “total emphasis” game, participants were instructed to focus evenly on all subcomponents of the task in order to achieve the highest total score. They were told that this was their first chance to earn an extra $30. After the game, a feedback screen listed their total score and subscores. Total score was read to the participant by the experimenter. We compared the stimulation montage groups on this task and found no group differences (see Results).

***Training***. During Games 2–10, participants completed four “emphasis change” (variable priority) games (Gopher et al., 1989) followed by five “total emphasis” games. Previous work has found that emphasis change instructions early in training—focus on one subcomponent while playing the whole game—lead to better SF performance (Gopher et al., 1989). tDCS was administered during training starting with Game 2 and ending after 30 min of timed stimulation during Game 9. Emphasis change games (2–5) each emphasized a different subcomponent of the overall game- participants were instructed to play all aspects of the game, but to focus their attention on one of the 4 sub-components; control, velocity, speed, or points. After each game, the score received on the respective subscore was reported to the participant from the feedback screen. The remainder of the training games (6–10) were “total emphasis,” with participants instructed to focus on all 4 components of the game equally. Total score was reported after each game.

***Training assessment***. Immediately after the 10th game, the participants performed another “total emphasis” game as a post-training assessment (Game 11) and were told that this would be their second chance to win $30. Participants were instructed to perform as well as they could on all aspects of the game. The total score was reported to the participant after the game.

***Assessment after 1-hr delay***. Next, participants were allowed to leave but instructed to come back to the testing room 1 h later to complete the study. During the 1 h break, participants were free to leave the building. After the hour, participants returned and completed a final “total emphasis” game (Game 12) on which they were again instructed to perform as well as they could on all aspects of the game. They were also informed that this was their final chance to win $30.

### tDCS parameters

Before participants started Game 2, electrodes were positioned on the head and connected to the stimulation unit (ActivaDose II, ActivaTek). Electrode placement followed the 10–20 EEG system. tDCS was delivered via two 2 × 2 in. electrodes fitted with saline soaked sponges (resulting in sponge contact area of 1.25 × 1.25 in.), with the anode placed over the target area and cathode placed on the contralateral upper arm. Use of an extracephalic reference has been investigated for its potential to influence brain stem autonomic functions. In a recent study, no differential effect of real or sham stimulation with an extracephalic reference was found on heart rate, respiratory rate, blood pressure, or sympathetic tone (Vandermeeren et al., 2010).

Participants were randomly assigned to receive either sham stimulation (0.2 mA with anode at F10) or 2 mA stimulation with anode placed at (a) C3, (b) C4, (c) F9, or (d) F10 (cathode on contralateral arm). Previous studies have demonstrated the safety of 2 mA stimulation on a range of tasks (Keeser et al., 2011; Chib et al., 2013; Clark and Parasuraman, 2014), including complex, real-world tasks (Fecteau et al., 2007; Clark et al., 2012; Falcone et al., 2012). Furthermore, studies comparing dosage levels have demonstrated performance modulation with 2 mA, but not with 1 mA (Boggio et al., 2006; Teo et al., 2011; Moos et al., 2012) or 0.6 mA (Clark et al., 2012) compared to sham. Participants were blinded as to whether they were administered active or sham stimulation.

Sham stimulation was administered by first ramping the current up to 2mA and then slowly decreasing the current to 0.2 mA where it remained. This ramp-up/ramp-down method for sham stimulation has been shown to be indistinguishable from active stimulation to the participants (Ambrus et al., 2012) and has been used in several previous studies (Fecteau et al., 2007; Nelson et al., 2014).

**Figure 2:**
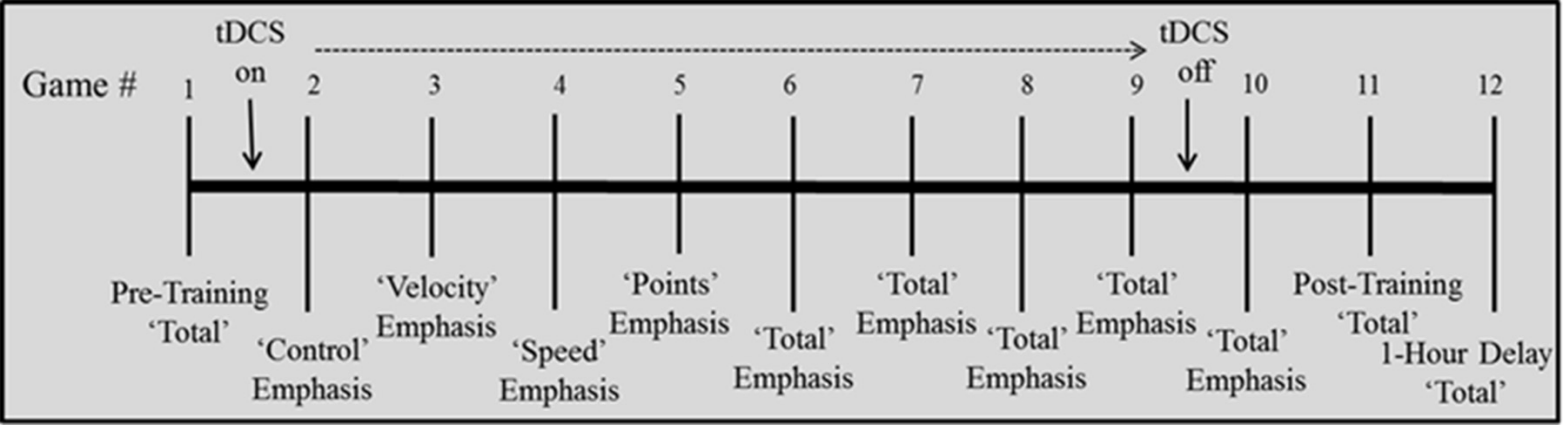
**Depiction of training schedule**.

The timed 30 min of stimulation automatically ended mid-way through Game 9 (the third total emphasis game). After completion of this game, the electrodes were removed and the participant completed the remainder of the training and post-training assessments without stimulation (see Figure 2). A mood questionnaire in which participants rated their current feelings of fatigue, boredom, and sadness was completed both before and after stimulation to assess any changes in affect due to stimulation. A sensation questionnaire was completed by participants at three timepoints (at ramp-up, after 5 min, and after 15 min of stimulation) during both active and sham stimulation. This required rating on a scale of 1–10 the degree of “itching,” “burning/heating,” and “tingling”-each assessed separately. Due to variability in the first assessment, which was given during the ramp-up (for active conditions) or ramp-up/ramp-down (during sham condition) period, sensation ratings from the latter two assessments were used to evaluate any potential effect of sensation on performance.

**Figure 3:**
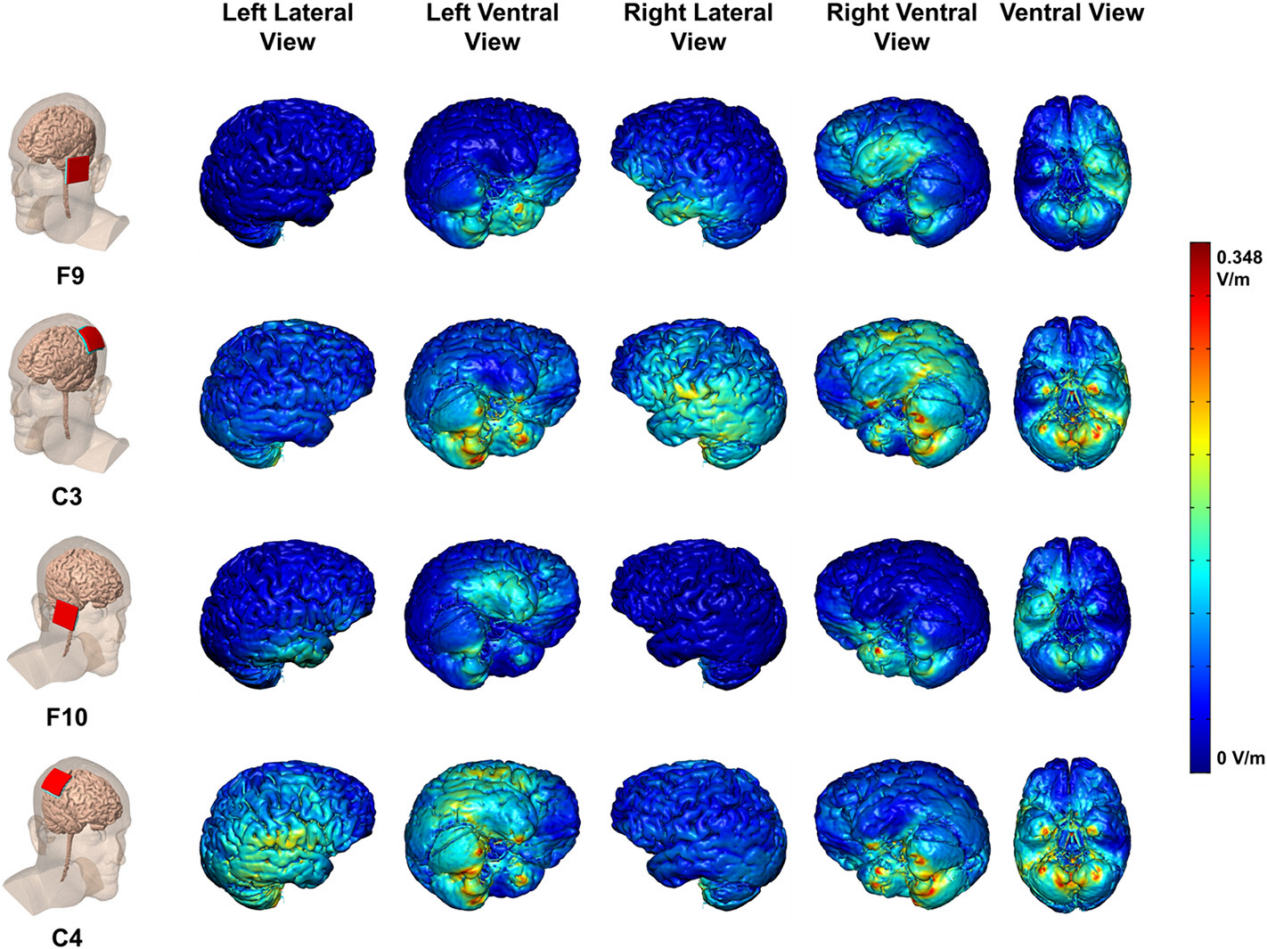
**Maps of electric field current flow over cortex for each stimulation montage with cathode on contralateral upper arm**. Colors indicate electrical field intensities in V/m per mA. Peak electrical field per 1 mA stimulation: 0.348 V/m per mA

Each stimulation montage (anode at F9, F10, C3, C4 to cathode on contralateral arm) was modeled with Finite Element Method (FEM) models to predict regional brain current flow (Figure 3). High-resolution head models were created from a previously segmented adult male based on a T1 MRI scan with a 1 mm isotropic resolution. Models of sponges and electrodes were positioned and resampled into the image volume before a voxel-based volumetric mesh was generated using ScanCAD and ScanIP (Simpleware Ltd., Exeter, UK). This mesh was imported into a FEM solver (COMSOL 3.5a, Dassault Systèmes Corp., Waltham, MA) modeling electrostatic physics. One of nine conductivities were assigned to the various materials: skin (0.465 S/m), fat (0.025 S/m), skull (0.01 S/m), cerebrospinal fluid (1.65 S/m), gray matter (0.276 S/m), white matter (0.126 S/m), air (1e-15 S/m), electrode (5.99e7 S/m), saline-soaked sponge (1.4 S/m). The field equation (Lapace, ∇ · (σ∇V) = 0) was solved with boundary conditions set to: insulated on the skin surface, ground on the cathode surface, inward current density on the anode surface (see details in Datta et al., 2009).

The FEM model for F9 and F10 anodes (cathode on contralateral arm) generates electrical fields in lateral and ventral aspects of the temporal and prefrontal cortices (Figure 3), with only minor spread to the ventral surface of the contralateral hemisphere. The FEM model for the C3 and C4 anodes (cathode on contralateral arm) generates electrical fields with a focus over the TPJ, and including lateral temporal and parietal cortices, largely within the stimulated hemisphere. Thus, current flow patterns were specific to the electrode montage (Bikson et al., 2010). Based on these models, we targeted networks with electrode locations as follows: (a) the dorsal attention network with C3 and C4 (left and right IPS, respectively); (b) the right hemisphere ventral attention network with F10 (right ventral prefrontal) and C4 (right TPJ). C4 may stimulate both right IPS and TPJ, as will be discussed below.

## Results

Of the 100 participants, 19 participants who were enrolled in the study were excluded for the following reasons. Data from 13 participants were excluded due to computer/experimenter error or not returning to complete the study, 6 participants were eliminated as outliers because they scored two or more standard deviations from the group mean on any three or more games using the total score measurement (3 were eliminated from sham, 2 from F10 and 1 from F9 groups). To compare montage groups, a One-Way Analysis of Variance (ANOVA) was conducted on each measure of demographic data including age, gender, and reported hours per week of first person shooter gameplay. There were no significant differences between the groups. There were also no significant differences between groups on performance of the aiming task score or of the pre-stimulation baseline game (Game 1). Thus, the randomly assigned groups did not differ on important factors, including ability to learn the task. Demographic information is listed in Table 1.

**Table 1:** **Demographics**.

**Stimulation group**	***n***	**Age**	**# (%) female**	**First person shooter game hours/wk**	**Aiming task score**	**Pre-training game total score**
Sham	14	20.50	13 (93%)	1.43	830.00	−3835.64
C3	18	20.06	12 (67%)	1.00	886.11	−3728.56
C4	19	21.94	11 (58%)	2.50	1236.84	−3263.47
F9	15	20.93	12 (80%)	0.90	1322.00	−3646.53
F10	15	22.00	12 (80%)	0.33	784.67	−3580.80
Total	81	21.31	60 (74%)	1.06	1020.62	−3595.42
Significance		ns	ns	ns	ns	ns

*Sample sizes and demographic information for each stimulation group*.

**Table 2:** **Standard errors for total score and subscores for each training game: pre-training total emphasis (pre-train.), control emphasis, velocity emphasis, speed emphasis, points emphasis, total emphasis, post-training total emphasis (post-train.) and 1-hr delay total emphasis game**.

**Game**	**Pre-train**.	**Control**	**Velocity**	**Speed**	**Points**	**Total**	**Total**	**Total**	**Total**	**Total**	**Post-train**.	**1-hr Delay**
**TOTAL SCORE**												
Sham	200.46	250.17	299.58	291.27	303.03	327.20	382.03	371.36	364.52	364.84	377.53	384.83
C3	176.79	220.63	264.20	256.88	267.25	288.56	336.92	327.51	321.47	321.76	332.95	339.38
C4	172.07	214.74	257.15	250.03	260.12	280.86	327.93	318.78	312.90	313.18	324.07	330.33
F9	193.66	241.68	289.42	281.40	292.75	316.10	369.08	358.77	352.16	352.47	364.73	371.78
F10	168.92	184.75	217.61	201.77	211.63	230.97	252.06	248.15	256.65	268.90	280.67	278.52
**CONTROL SUBSCORE**												
Sham	111.20	168.90	188.35	174.41	186.87	180.12	192.48	188.18	195.31	195.15	197.91	202.85
C3	98.06	148.95	166.11	153.82	164.81	158.85	169.75	165.96	172.25	172.11	174.54	178.90
C4	95.45	144.98	161.68	149.71	160.41	154.62	165.23	161.53	167.65	167.52	169.89	174.13
F9	107.42	163.17	181.96	168.50	180.54	174.02	185.96	181.80	188.69	188.54	191.20	195.98
F10	107.42	163.17	181.96	168.50	180.54	174.02	185.96	181.80	188.69	188.54	191.20	195.98
**VELOCITY SUBSCORE**												
Sham	74.99	74.13	113.42	108.08	110.78	113.69	119.44	146.66	142.78	147.07	142.45	146.97
C3	66.14	65.38	100.03	95.32	97.70	100.26	105.34	129.34	125.92	129.71	125.63	129.61
C4	64.37	63.63	97.36	92.78	95.09	97.59	102.53	125.89	122.56	126.25	122.28	126.16
F9	72.45	71.62	109.58	104.42	107.02	109.83	115.39	141.69	137.93	142.09	137.62	141.98
F10	72.45	71.62	109.58	104.42	107.02	109.83	115.39	141.69	137.93	142.09	137.62	141.98
**SPEED SUBSCORE**												
Sham	44.88	43.70	45.35	60.86	47.42	47.81	53.42	49.59	58.88	45.20	52.74	52.13
C3	39.58	38.54	39.99	53.68	41.82	42.17	47.11	43.73	51.93	39.86	46.51	45.97
C4	38.52	37.52	38.93	52.25	40.70	41.04	45.85	42.57	50.55	38.80	45.27	44.75
F9	43.36	42.22	43.81	58.80	45.81	46.19	51.61	47.91	56.89	43.67	50.95	50.36
F10	43.36	42.22	43.81	58.80	45.81	46.19	51.61	47.91	56.89	43.67	50.95	50.36
**POINTS SUBSCORE**												
Sham	176.40	151.10	150.54	138.48	175.80	145.06	172.73	176.51	165.75	178.97	170.53	177.51
C3	155.57	133.26	132.77	122.13	155.04	127.93	152.34	155.67	146.18	157.84	150.39	156.55
C4	151.42	129.70	129.23	118.87	150.90	124.52	148.27	151.51	142.28	153.63	146.38	152.38
F9	170.42	145.98	145.44	133.79	169.83	140.14	166.88	170.52	160.13	172.90	164.75	171.50
F10	170.42	145.98	145.44	133.79	169.83	140.14	166.88	170.52	160.13	172.90	164.75	171.50

Participants tended to feel a tingling/itching sensation during stimulation onset, as reported in previous studies (Nitsche et al., 2003; de Vries et al., 2009; Chib et al., 2013). Each sensation measure (itching, heating, tingling) was examined in a univariate ANOVA, revealing significant main effects of group for heating [*F*_(4, 74)_ = 3.91, *p* = 0.006] and tingling [*F*_(4, 74)_ = 3.80, *p* = 0.007], but not for itching. *Post-hoc* pair-wise comparisons (Tukey HSD) revealed that for heating, the only significant group difference was between sham and F10 (*p* = 0.01). There were no significant pair-wise differences for tingling. To assess the effect of sensation on performance, each sensation measure was correlated with performance [the average total score from the two post-training assessment games (Games 11 and 12)]. There were no significant effects. There was no significant group effect of mood change scores (score before stimulation minus score after stimulation) as assessed by a univariate ANOVA.

Total score (composed of the sum of the 4 subscores) was evaluated with a mixed-design omnibus ANOVA, with planned follow-up ANOVAs on each of the four subscores. Game was the within-subjects factor and stimulation group was the between-subjects factor. When the assumption of sphericity was violated on Mauchley's test, degrees of freedom were adjusted using Huynh-Feldt correction. For significant main effects of stimulation group, *post-hoc* pair-wise comparisons were computed using Tukey's test.

As described above, previous studies that trained SF over weeks have used the aiming task either as an exclusionary factor or as a covariate (e.g., Blumen et al., 2010). In our data, when included as a covariate, aiming score interacted significantly with the games factor, making it unsuitable as a covariate. Another covariate previously used in SF studies is the baseline game for each subtask, administered before stimulation begins (e.g., Lee et al., 2012). In our data, when the baseline game was included as a covariate, Group × Game interactions were weaker and power of each group main effect was reduced compared to the same data set analyzed without a covariate. Therefore, we analyzed without a covariate, using the baseline game as the first repeated-measure. Nevertheless, it is important to note that the results with and without a covariate were very similar. For the velocity analysis, with the covariate included there was a main effect of group with no interaction. There were significant pairwise differences between C4 and all other stimulation groups (*post-hoc* pair-wise comparisons, Bonferroni corrected). Without the covariate included, the Group x Game interaction was significant and simple main effects showed that C4 stimulation improved performance compared to the other active stimulation groups on Game 3 to Game 11. Participants were randomly assigned to stimulation groups.

### Omnibus analysis

**Figure 4:**
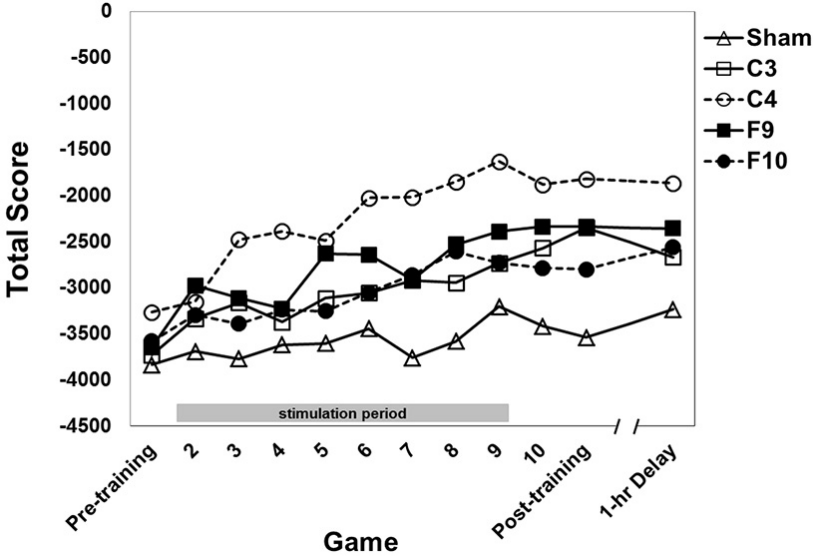
**Total score (sum of 4 subscores) for each stimulation group across 12 games with varying instructional emphasis**. Pre-training, Post-training and 1-hr Delay games were all “total emphasis” games. The training block consisted of Games 2 through 10. Game 2 was “control score” emphasis, Game 3 was “velocity score” emphasis, Game 4 was “speed score” emphasis, Game 5 was “points score” emphasis and Games 6–10 were “total score” emphasis. The 1-hr delay game began 1 hour after the end of the post-training game. Timed stimulation started prior to Game 2 and ended during Game 9 (gray bar). Standard errors are listed separately in Table 2.

**Figure 5:**
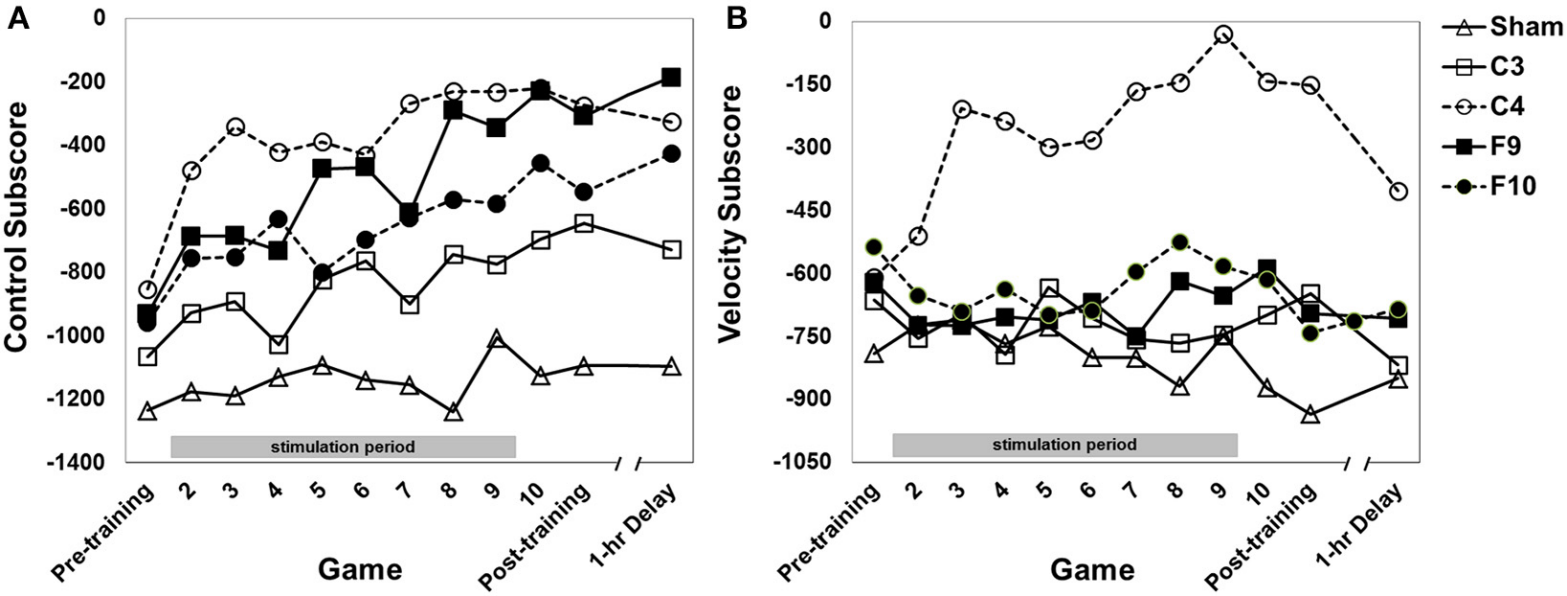
**(A)** Control subscore for each stimulation group across 12 games with varying instructional emphasis. **(B)** Velocity subscore for each stimulation group across all 12 games with varying instructional emphasis. Pre-training, Post-training and 1-hr Delay games were all “total emphasis” games. The training block consisted of Games 2 through 10. Game 2 was “control score” emphasis, Game 3 was “velocity score” emphasis, Game 4 was “speed score” emphasis, Game 5 was “points score” emphasis and Games 6–10 were “total score” emphasis. The 1-hr delay game began 1 hr after the end of the post-training game. Timed stimulation started prior to Game 2 and ended during Game 9 (gray bar). Standard errors are listed separately in Table 2. For the velocity subscore, Table 3 shows results of a simple effects analysis of group at each level of game.

**Figure 6:**
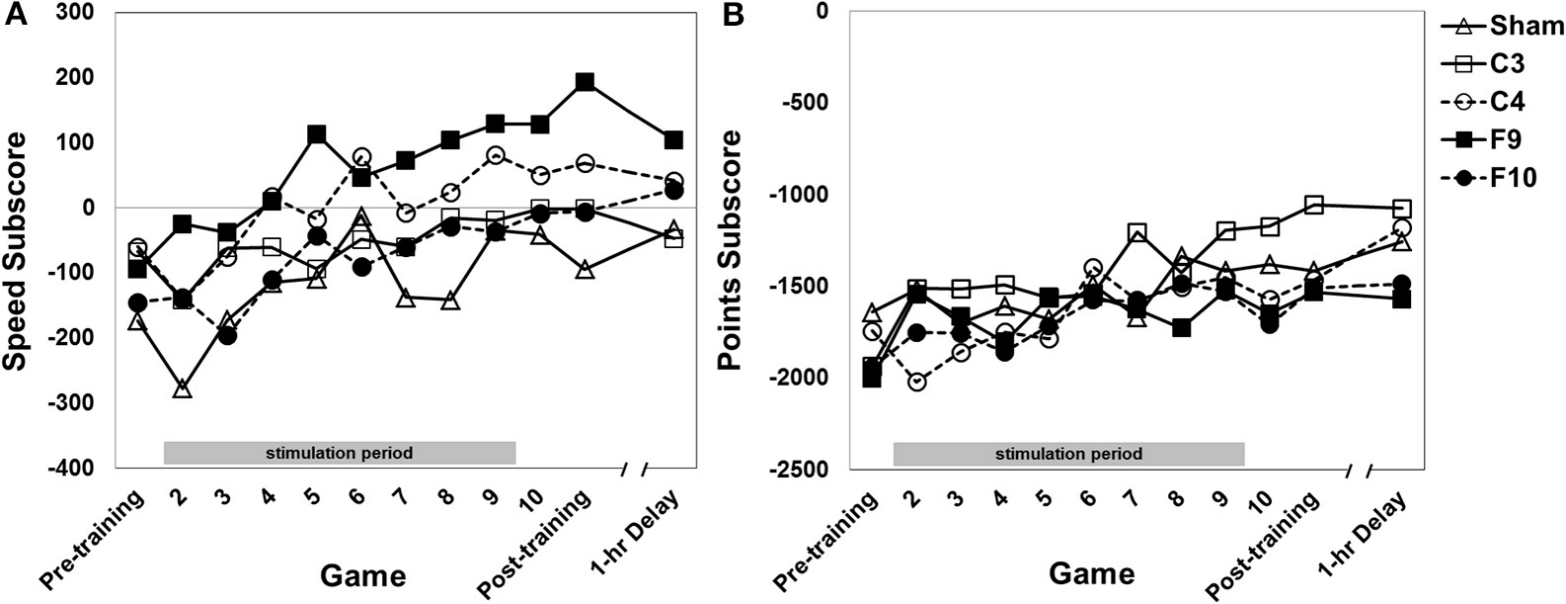
**(A)** Speed subscore for each stimulation group across 12 games with varying instructional emphasis. **(B)** Points subscore for each stimulation group across all 12 games with varying instructional emphasis. Pre-training, Post-training and 1-hr Delay games were all “total emphasis” games. The training block consisted of Games 2 through 10. Game 2 was “control score” emphasis, Game 3 was “velocity score” emphasis, Game 4 was “speed score” emphasis, Game 5 was “points score” emphasis and Games 6–10 were “total score” emphasis. The 1-hr delay game began 1 hour after the end of the post-training game. Timed stimulation started prior to Game 2 and ended during Game 9 (gray bar). Standard errors are listed separately in Table 2.

An omnibus mixed-design ANOVA was conducted on total score with stimulation group as the between subjects factor and game as the within subjects factor. Typically all subscores are negative during the first sessions of training before becoming positive (e.g., Gopher et al., 1989; Prakash et al., 2012). Since total score is the sum of those scores, it is more negative than the others (Figures 4–**6**). Total score performance improved over training games (Figure 4, main effect of game [*F*_(9.71, 727.89)_ = 17.75, *p* < 0.0001, partial eta squared = 0.027]. The groups differed significantly from each other [*F*_(4, 75)_ = 3.61, *p* = 0.009, partial eta squared = 0.151]. *Post-hoc* pairwise comparisons between stimulation groups showed that the C4 group showed better performance than the sham group (*p* = 0.004). Group × Game was not significant.

### Planned analyses of subscores

Because we hypothesized that stimulation aimed at dorsal and ventral networks would differentially influence performance of the subtasks (control, velocity, speed and points), we planned separate analyses of each subscore. Each subscore is described in the Methods. Stimulation group was the between subjects factor while game was the within subjects factor in each of the following analyses.

#### Control

Performance on the control subscore (fly the spaceship within a fixed hexagonal area surrounding the fortress) improved over games of training (Figure 5A, main effect of game [*F*_(7.40, 555.12)_ = 12.43, *p* < 0.0001, partial eta squared = 0.036]. The stimulation groups differed overall [main effect of group, *F*_(4, 75)_ = 3.94, *p* = 0.006, partial eta squared = 0.163]. *Post-hoc* pair-wise comparisons between groups (Tukey HSD) showed that both the C4 and the F9 groups performed significantly better than the sham group (*p* = 0.005 and *p* = 0.040, respectively). No other pair-wise comparisons were significant. The interaction of Group × Game was not significant.

#### Velocity

Performance on the Velocity subscore (fly the spaceship with slow, controlled movements) was affected by stimulation group [Figure 5B, main effect of group, *F*_(4, 75)_ = 5.90, *p* < 0.0001, partial eta squared = 0.239]. The effect of group was modified by a Group × Game interation [*F*_(30.40, 570.02)_ = 1.78, *p* = 0.014, partial eta squared = 0.075] indicating that the performance of the groups changed differentially over games. Simple main effects comparing groups at each level of game found that groups differed significantly on all games except for Games 1, 2, and 12. Game 1 was a baseline game before stimulation and Game 2 was the first stimulated game. Game 12 was played about 1 h after training and stimulation ended. On Games 3 through the post-training game C4 stimulation improved performance compared to sham stimulation. C4 stimulation also improved performance compared to the other active stimulation groups (C3, F9, and F10) on various other games (see Table 3).

**Table 3:** **Velocity subscore simple effects comparing group at each game**.

**Game**			**Sham**	**C3**	**F9**	**F10**
Pre-training	Total emphasis	C4 >	ns	ns	ns	ns
2	Control emphasis	C4 >	ns	ns	ns	ns
3	Velocity emphasis	C4 >	**0.012**	**0.008**	**0.007**	**0.015**
4	Speed emphasis	C4 >	**0.005**	**0.001**	**0.013**	0.054
5	Points emphasis	C4 >	**0.046**	ns	0.053	0.068
6	Total emphasis	C4 >	**0.009**	**0.033**	ns	0.071
7	Total emphasis	C4 >	**0.001**	**0.001**	**0.003**	0.067
8	Total emphasis	C4 >	**0.004**	**0.009**	ns	ns
9	Total emphasis	C4 >	**0.003**	**0.001**	**0.011**	**0.036**
10	Total emphasis	C4 >	**0.003**	**0.029**	ns	ns
Post-training	Total emphasis	C4 >	**0.001**	0.059	**0.042**	**0.020**
1-hr Delay	Total emphasis	C4 >	ns	ns	ns	ns

*P-values listed are for significant effects and effects with a non-significant trend. Bolded values indicate p < 0.05*.

#### Speed

Performance on the speed subscore (speed and accuracy in dealing with mines) improved over games of training [Figure 6A, main effect of game, *F*_(11, 825)_ = 10.54, *p* = 0.0001, partial eta squared = 0.034]. The stimulation groups differed [main effect of group, *F*_(4, 75)_ = 6.22, *p* > 0.0001, partial eta squared = 0.191]. *Post-hoc* pair-wise comparisons of group effects (Tukey HSD) revealed F9 stimulation resulted in a higher speed subscore compared to these stimulation groups: (a) sham (*p* < 0.0001); (b) F10 (*p* = 0.009); (c) C3 (*p* = 0.023). C4 group performance was greater than sham (*p* = 0.019). The interaction of Group × Game was not significant.

#### Points

Performance on the points subscore (based on shooting and destroying the fortress while protecting the ship from the fortress) improved over games of training [Figure 6B, main effect of game, *F*_(10.60, 795.28)_ = 7.21, *p* < 0.0001, partial eta squared = 0.026]. There was no effect of group, nor an interaction effect.

## Discussion

We obtained partial support for our hypothesis that tDCS aimed at dorsal and ventral attention networks exerts selective effects on the initial stage of multi-task training. While our results are consistent with the growing literature showing the importance of dorsal and ventral attention networks in training, they were more complex than we predicted. The strongest effects on training were seen when the anode was over right parietal cortex (C4) during two of the four subtasks—flying the ship within the hexagons (control subtask) and operating the ship with slow, controlled movements (velocity subtask). The benefits of the C4 anode were strong enough to be reflected in the sum of all subscores (total score) and were evident very early in training. In the velocity subscore, we found that effects of right parietal stimulation were evident after only the second stimulated game. On the other hand, the speed subtask (memory for irregularly-appearing symbols that change game-to-game) benefited from an anode over left prefrontal cortex (F9). No stimulation montage affected the points subtask.

These findings suggest that initial learning of a complex multi-task engages attention and working memory systems simultaneously, with these systems separately stimulated by specific tDCS montages. That one montage (C4 to left shoulder) improved performance on three of the four subtasks as well as total performance shows that tDCS may be practical for use in real world multi-task training where it would be desirable to use only one montage.

Confidence in our results is increased by (a) the absence of a difference between the groups before stimulation, (b) the evident learning on each subtask during the abbreviated training (e.g., both the initial level and the 1-session increase in the control subscore we observed were comparable to those reported for the same task by Lee et al., 2012), and (c) by the differential effects of montage.

### Control and velocity subtasks

We had hypothesized that control and velocity subtasks (both involved flying the spaceship) would benefit selectively from stimulating the dorsal attention network (anodes at C3 and C4). This was based on literature showing alteration in the dorsal attention network with cognitive training (e.g., Lewis et al., 2009; Takeuchi et al., 2010; Strenziok et al., 2014), and based on evidence that tDCS selectively increased activity in dorsal attention networks (Keeser et al., 2011). Contrary to that prediction, we found that control and velocity subscores (and total score) benefited from C4 but not C3 stimulation. Moreover, for the velocity subscore this effect increased as stimulated training proceeded. Simple effects testing revealed that the effects of C4 stimulation were strongest after stimulation had started and several games had been played (Table 3).

Why did right parietal stimulation selectively affect ability to control the ship? We consider several explanations.

- Was the effect of C4 stimulation due to altered motor cortex excitability? C4 lies over the right central sulcus (near the motor strip) and tDCS over C3 and C4 can heighten motor cortex excitability (reviewed in Jacobson et al., 2012). However, control and velocity measures both depend on fine movements of the joystick in the right hand which is controlled by left hemisphere motor cortex (C3). Therefore, our findings of a benefit from a C4 montage are not consistent with a motor explanation.
- Was the effect of C4 stimulation due to increased activity in the right hemisphere ventral attention network? If our results were due simply to activation of that network, then benefits would have been seen with stimulation of right ventral prefrontal (F10)—an established node in the ventral attention network (Corbetta et al., 2008). However, we observed no effect of stimulating F10.
- Was the effect of C4 stimulation due to simultaneous activation of both dorsal and ventral attention networks? According to the Corbetta et al. (2008) model, when visuospatial attention is focused while the dorsal attention network is active, the occurrence of a new, task-relevant event activates the ventral attention network *together with* the dorsal attention network. FEM modeling (Figure 3) shows that an anode at C4 (cathode on left shoulder) induces an electric field that includes right IPS (dorsal network) and right TPJ (ventral network). Therefore, the C4 montage could have stimulated nodes in both dorsal and ventral attention networks. If stimulation of the dorsal attention network alone facilitated flying the ship, then a C3 anode would have been as effective as a C4 anode. That was not observed. If stimulation of the ventral attention network alone facilitated flying the ship, then an F10 anode would have been effective. That was not observed. We speculate that simultaneous activation of dorsal and ventral attention networks by C4 anode stimulation while flying the ship (control, velocity subtasks) facilitated efficient redirection of attention to sudden onset events (mines, points subtasks) with less cost to flying the ship compared to other montages. This speculation is consistent with Corbetta's hypothesis about the way that dorsal and ventral attention networks operate. Our results add to the growing literature (reviewed above) of the importance of dorsal and ventral attention networks in training.

We also observed a benefit of F9 stimulation on the control subscore, but not the velocity subscore. F9 lies over left ventral frontal cortex, not associated with either dorsal or ventral attention networks. This result is not consistent with our hypothesis.

### Speed and points subtasks

A different pattern of results was seen with the other two subtasks—speed and points. We had hypothesized that because processing irregularly-timed speed and points symbols would require redirection of attention from flying the ship to processing those symbols, the need to intermittently respond to symbols while flying the spaceship would benefit from stimulation of the right hemisphere ventral network. On the points subscore, we found no effects of tDCS. On the speed subscore, the C4 anode group performed better than the sham. The latter could be consistent with an attentional explanation, but also with a motor explanation as the left hand responded. The speed measure depends on (a) remembering which three letters had been displayed at the start of each game to indicate which mines were designated “foe” mines for that game, and (b) taking appropriate action when mines suddenly appeared. The speed measure also showed benefits of a left ventral PFC anode (F9), insofar as that group performed better than the other groups, except for C4. We speculate that the working memory load associated with retaining and updating memory of “foe” mines from game to game may have benefited from stimulation of left PFC, previously found to benefit working memory (e.g., Hoy et al., 2013; Meiron and Lavidor, 2013). However, both left and right PFC stimulation at F3 and F4 have been found to have other effects, e.g., more cautious driving in a simulator (Beeli et al., 2008) and reduced risk taking (Fecteau et al., 2007).

### Cognitive training and attention networks

Our finding that multi-task cognitive training benefited from right parietal stimulation is consistent with the growing evidence that attention networks are important in cognitive training. The dorsal attention network has been consistently found to undergo structural and/or functional change following training on juggling (Draganski et al., 2004; Scholz et al., 2009), working memory (Takeuchi et al., 2010), visual search (Lewis et al., 2009), and auditory perception (Strenziok et al., 2014). Two studies observed that reliance on the dorsal attention network decreased as perception improved following perceptual training (Lewis et al., 2009; Strenziok et al., 2014). The ventral attention network has also been implicated in SF training (Lee et al., 2012; Prakash et al., 2012; Strenziok et al., 2014). Those findings are consistent with the literature showing a decreased reliance on control and attentional processing after hours or days of training (reviewed in Kelly and Garavan, 2005). Considered together, this evidence suggests the importance of stimulating very early in training—as in the present study—when visuospatial attention is most active (Kelly and Garavan, 2005; Strenziok et al., 2014) and when plasticity mechanisms favor conversion from short-term to long-term memory (Jones et al., 2001; Maroteaux et al., 2014).

This evidence from the present study that right parietal stimulation selectively facilitated learning of control, velocity, and speed subtasks of the SF multi-task leads to the interpretation that simultaneous stimulation of dorsal and ventral attention networks benefited SF training because of increased efficiency in redirecting visuospatial attention from one subtask to another. Our findings are also broadly consistent with previous work from Kramer's lab showing that SF training led to reduced activation of ventral frontal regions (a node in the ventral attention network) after training (Lee et al., 2012; Prakash et al., 2012). Those two studies assessed activation patterns at the end of training. We modulated activation at the beginning of training. To the extent that SF training requires frequent reorienting of visuospatial attention, the ventral attention network may be important early in training—producing our results—but become less important as the task is learned—leading to the reduced activation of the ventral attention network seen after SF training in Kramer's studies (Lee et al., 2012; Prakash et al., 2012).

How do we reconcile our findings of the benefits of simultaneous stimulation of dorsal and ventral attention networks on ship control measures with findings from other training tasks found to induce changes specifically in the dorsal attention network (Lewis et al., 2009; Lövdén et al., 2010; Takeuchi et al., 2010; Strenziok et al., 2014)? We speculate that cognitive training that does not require multi-tasking (e.g., the perception training tasks used by Lewis et al., 2009 and Strenziok et al., 2014) may rely on focused attention and hence on the dorsal attention network. On the other hand, training that does require multi-tasking, like SF, may rely more on the ability to redirect attention controlled by the ventral attention network (Lee et al., 2012; Prakash et al., 2012), found to be activated in conjunction with the dorsal attention network (Corbetta et al., 2008). It should be noted that while this explanation based on the role of attention in cognitive training is consistent with findings from ship control and velocity subscores, it does not explain the effectiveness of left ventral PFC (F9) stimulation for the speed measure.

## Conclusions and limitations

The present findings argue for development of tDCS as a training aid useful in real world settings where multi-tasking is critical. That we observed benefits of tDCS very early in training on a task typically mastered over several weeks shows the practicality of using tDCS early in real world training. That we observed selective effects of montage—on ship flying with a C4 anode and on speed (friend or foe) management with an F9 anode—shows the importance of selecting a montage based on empirical evidence from the specific task. That we observed effects of stimulation consistent with activation of dorsal and ventral attention networks is important for understanding brain mechanisms of multi-task training and ways to heighten them. Although our interpretation based on attention networks may be wrong, it is rooted in a theory (Corbetta et al., 2008) and has empirical support in the training literature. Further, our interpretation is consistent with the growing evidence that cognition emerges from the concerted function of brain areas working in large-scale networks (Bressler and Menon, 2010). As such, our interpretation can be considered a parsimonious, theory-based account of our results.

Although we observed benefits of tDCS early in training, it is possible they would be similar or even greater with stimulation later in training or throughout training. Questions of timing of tDCS stimulation have not yet been addressed. That is important in light of evidence that mechanisms early in learning are different from those late in learning (e.g., Kelly and Garavan, 2005). Another limitation concerns the relatively large electric fields induced by the sponge electrodes (Kuo et al., 2013). Yet, despite the size of the electric fields, the few other studies that have compared different montages have likewise found very montage-specific cognitive effects, e.g., benefits of left but not right parietal tDCS on mental arithmetic (Hauser et al., 2013). Future work using “high definition” electrodes which induce smaller electric fields (Datta et al., 2009) or using arrays of small electrodes (e.g., Dmochowski et al., 2011) may be able to target resting state networks more precisely. Neuroimaging studies will be needed to confirm the activated brain regions and determine effects of specific tDCS montages on functional connectivity. Ultimately, however, the most important evidence regarding effects of tDCS brain stimulation on multi-task learning is the effect on subtask performance.

### Conflict of interest statement

The authors declare that the research was conducted in the absence of any commercial or financial relationships that could be construed as a potential conflict of interest.
